# Unisexual reproduction in the global human fungal pathogen *Cryptococcus neoformans*

**DOI:** 10.1101/2025.06.02.657540

**Published:** 2025-06-03

**Authors:** Sheng Sun, Zhuyun Bian, Ziyan Xu, Yeseul Choi, Joseph Heitman

**Affiliations:** Department of Molecular Genetics and Microbiology, Duke University Medical Center, Durham, North Carolina, USA

**Keywords:** fungi, sexual development, Ric8, guanine nucleotide exchange factor (GEF), G protein

## Abstract

Sexual reproduction is a central tenet of the eukaryotic life cycle, essential for the long-term survival of a species. Sex promotes genetic diversity and facilitates natural selection, and in eukaryotes such as unicellular fungi, sexual reproduction leads to hyphal growth and spore production, enabling escape from harsh environments and long-distance dispersal. The human fungal pathogen *Cryptococcus* species complex (encompassing *C. neoformans*, *C. deneoformans*, and the *C. gattii* sub-complexes) exhibit diversity in sexual reproduction, including α-**a** mating, pseudosexual reproduction, as well as unisexual reproduction initiated from a single isolate or between isolates of the same mating type. A central conundrum is that while most *Cryptococcus* natural populations exhibit significant α mating type bias, genetic and genomic analyses show recombination occurs in nature. The discovery of unisexual reproduction in *C. deneoformans* provided insight; however, thus far unisexual reproduction has never been observed in the predominant global pathogenic species *C. neoformans*. Here, we provide evidence that mutating the *RIC8* gene, which encodes a conserved guanine nucleotide exchange factor (GEF) involved in activation of Gα proteins, enables unisexual reproduction in *C. neoformans*. Additionally, we show that genetic variation in the natural population promotes unisexual reproduction, and unisexual reproduction in *C. neoformans* shares similarities with that in *C. deneoformans* and involves canonical meiotic recombination. Finally, our data suggest that Ric8 interacts with Gpa1 and Gpa2 during α-**a** mating, but the localization of these Gα proteins is not influenced by Ric8 during unisexual reproduction. This suggests differential regulation of the Gα proteins, likely involving the Ric8 protein, could underly switch between different modes of sexual reproduction in *Cryptococcus*. Our study further highlights that the highly conserved Ric8 GEF can act as an important regulator of cellular development in response to environmental stimuli and modulates sexual reproduction in nature. We hypothesize that unisexual reproduction occurs much more frequently in nature than currently appreciated, and possibly in other fungal species as well.

## Introduction

Sexual reproduction is a fundamental process in eukaryotes. Through meiosis, sex can produce progeny population with increased genetic diversity and phenotypic variation, therefore facilitating natural selection and playing a critical role in the long-term survival of a species. Sexual reproduction is of particular importance to unicellular eukaryotes such as fungi. While most fungal species typically reproduce mitotically in nature, they are susceptible to changing environments such as fluctuations in temperature, nutrients, antifungal natural products, as well as co-inhabitants in their natural niches. Sexual reproduction allows generation of recombinant progeny with higher fitness in the current environment, as well as production of sexual spores that are better equipped for dispersal and withstanding harsh environments. Additionally, for yeast species, sexual reproduction can be linked with hyphal growth, which can facilitate foraging for nutrients and escaping from unfavorable conditions. Fungi exhibit extraordinary diversity in the modes of sexual reproduction, including α-**a** mating, unisexual reproduction, parasexual reproduction, and pseudosexual reproduction (1–6).

One example of this reproductive plasticity can be found in the human fungal pathogen *Cryptococcus* species complex, which are basidiomycetous yeasts that cause pulmonary infections, as well as systemic infections leading to meningoencephalitis. Composed of three sub-species complexes, *C. neoformans*, *C. deneoformans*, and *C. gattii*, the pathogenic *Cryptococcus* species collectively cause over 220,000 infections globally each year, accounting for ~181,000 deaths annually (7). There are two mating types in *Cryptococcus*, α and **a**, defined by a single mating type locus (*MAT*) in the genome, and sexual reproduction between α and **a** cells (α-**a** sexual reproduction) of *Cryptococcus* were first characterized in the laboratory ~50 years ago (8–10).

Among natural *Cryptococcus* isolates, there is a significant bias toward the α mating type (>99% in some cases), and populations with balanced α and **a** mating types have only been found in a few restricted geographic areas (e.g. sub-Saharan Africa) (11). Nevertheless, population genetics and genomics studies indicate that most natural population of *Cryptococcus* are recombining, suggesting sexual reproduction is ongoing, even in populations with few, or no, *MAT***a** isolates (12). How a recombining population with highly unbalanced mating types is maintained has long puzzled researchers.

Some 20 years ago, Lin et al. (13) discovered a novel mode of sexual reproduction in *C. deneoformans*, termed unisexual reproduction. In this case, sexual reproduction can be initiated by either a single cell (through endoreplication) or two cells of the same mating type (through conjugation) and then undergo sexual development similar to α-**a** sexual reproduction, with hyphal growth, basidium formation, meiosis, and sporulation. Subsequently, unisexual reproduction has been characterized in other fungal species such as *Candida albicans* (14). Unisexual reproduction could potentially explain the observed mating type imbalance observed in *Cryptococcus* natural populations. However, until now it has only been well characterized in *C. deneoformans*. Recently, we showed that in *C. deneoformans* Ric8 is involved in regulating cell morphological developments, and mutation of the *RIC8* gene enables cells to undergo titanization and sexual development in solo cultures under mating inducing conditions (15). Interestingly, we also discovered that in the *C. deneoformans* strain XL280, which undergoes robust unisexual reproduction, harbors a nonsense mutation in the *RIC8* gene (13), indicating that Ric8 suppresses unisexual reproduction.

Ric8 (resistance to inhibitors of cholinesterase 8) and its homologs are conserved in a wide range of eukaryotes, from unicellular fungi to invertebrates and mammals. Functioning as both a non-receptor guanine nucleotide exchange factor (GEF) and a molecular chaperone, Ric8 catalyzes the exchange of GDP for GTP on Gα subunits and promotes their proper folding and membrane association (16–20). In contrast to G protein-coupled receptors (GPCRs), Ric8 lacks a transmembrane domain and operates entirely within the cytoplasm to modulate Gα activity (21, 22), similar to a group of other nonreceptor proteins that contain the “Gα-binding and -activating (GBA) motif” (23). In fungi, Ric8 regulates G-protein signaling required for the nematode-trapping lifecycle of *Arthrobotrys oligospora* (24). In animals, Ric8 functions as an intracellular protein that plays a pivotal role in the regulation of heterotrimeric G protein signaling, and is involved in a variety of key cellular processes such as cell polarity establishment, cytokinesis, and nervous system development, underscoring its essential role in embryogenesis and neurodevelopment (25). For example, in *Caenorhabditis elegans* Ric8 acts as a regulator of asymmetric cell division and centrosome dynamics (26). In mammals, Ric8 is expressed in two functionally distinct isoforms, Ric8A and Ric8B, that exhibit selective binding and activation profiles toward specific Gα subsets, and regulate distinct G protein signaling pathways, with Ric8A being associated with embryonic development and central nervous system formation through Gα_i_ and Gα_q_ signaling, and Ric8B involved in olfactory signaling and neural development via activation of Gα_s/olf_ (20–22, 26). Despite the partial overlap in their target Gα subunits, Ric8A and Ric8B serve non-redundant and essential functions in vivo, with complete deletion of either gene resulting in embryonic lethality in mice. Additionally, Ric8A and Ric8B deletion result in severe depletion of specific Gα proteins, suggesting that both isoforms are required for proper Gα protein maturation (22).

In *Cryptococcus*, studies have provided evidence that Ric8 interacts physically and functionally with two of the three Gα proteins, Gpa1 and Gpa2, but not Gpa3, and plays an important role in regulation of virulence related traits such as melanin and capsule production, as well as response to mating pheromones (27), and is required for titanization in *C. neoformans* (28). It is possible that Ric8, functioning through binding and activating the Gα proteins, could influence conserved GPCR pathways that transmit environmental stimuli, thus modulating the downstream signaling pathways involved in morphological development in *Cryptococcus*. This is consistent with our findings that Ric8 represses morphological development (selfing and titanization) in *C. deneoformans* (15). We hypothesized that Ric8 plays a similar role in repressing morphological development, including unisexual reproduction, in *C. neoformans*. Here, we show that mutating the *RIC8* gene induces the *C. neoformans* lab strain H99 to undergo unisexual reproduction. Additionally, we generated strains with enhanced unisexual fertility by crossing H99 *ric8* mutants with natural isolates, revealing the presence of variation promoting unisexual reproduction in nature. With the robust unisexually fertile strains, we elucidated the signaling pathways required for unisexual reproduction in *C. neoformans* and show that sporulation during *C. neoformans* selfing involves canonical meiosis. Our findings further highlight the diverse approaches that pathogenic *Cryptococcus* species utilize to accomplish sexual reproduction, with implications for their ecology, population genetics, epidemiology, and evolution.

## Results

### Deletion of *RIC8* enables unisexual reproduction in *C. neoformans* lab strains

**Figure 1.**
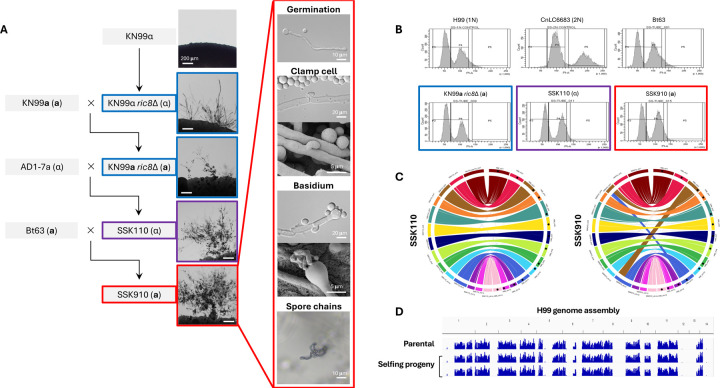
Haploid *C. neoformans* strains that undergo robust unisexual reproduction. (A) Genealogy of strains involved in constructing *C. neoformans* isolates that undergo robust unisexual reproduction, with light microscopy images next to the strains illustrating sexual structures on MS medium. Highlights with blue, purple, and red boundaries indicate low, medium, and high unisexual development, respectively. For strain SSK910 that undergoes the most robust unisexual reproduction, additional images for germination tube, clamp cell, basidium, and basidiospore chains are provided. (B) FACS analysis reveals unisexually fertile strains are haploid. (C) Circos plots of strains SSK110 (left) and SSK910 (right) reveal their genomes are colinear with the *C. neoformans* reference strain H99. For each plot, the right half shows chromosomes 1 to 14 (clockwise) of strain H99, and the left chromosomes 1 to 14 (counterclockwise) of strains SSK110 or SSK910. The discrepancies between strains SSK910 and H99 involving chromosomes 3 and 11 correspond to the reciprocal translocation between the two chromosomes in H99 as reported previously (48). (D) WGS and variant analysis of strain SSK910 and basidiospores dissected from SSK910 are depicted. Sequencing reads were mapped, and variants called with strain H99 as reference; blue bars indicating sequences that differ from H99. Both SSK910 and its progeny have identical genomes that are recombinant between H99 and non-H99 sequences (inherited from the natural isolates AD1-7a and Bt63 during construction of strain SSK910).

In a recent study, we discovered that Ric8, a guanine nucleotide exchange factor (GEF), acts as a suppressor of non-yeast form morphological development (e.g. titan cells and unisexual reproduction) in *C. deneoformans* under conditions inducing unisexual reproduction (15). Interestingly, the well characterized *C. deneoformans* isolate XL280, which undergoes highly robust unisexual reproduction, contains a nonsense mutation in the *RIC8* gene, rendering it non-functional. Taking this lead, we hypothesized that by deleting the *RIC8* gene, unisexual reproduction might be induced in the predominant global pathogenic species *C. neoformans*. To test this, the *RIC8* gene was deleted in the *C. neoformans* lab strain KN99α to generate KN99α *ric8*Δ::*NAT* mutant strains (SSI867 and SSI868, Supplemental Table S1). When grown under mating inducing conditions, structures resembling sexual development, including hyphae, basidia, and basidiospores were observed (Figure 1). Basidiospores were dissected from five individual basidia produced by strain SSI867 and analyses revealed: 1) spores from all of the basidia had high germination rates, 2) all of the spores harbored the NAT-resistant *RIC8* deletion, and 3) all of the basidiospores were *MAT*α (Table 1). This ruled out the possibility of accidental contamination by *MAT***a** cells during mating set-up, consistent with the conclusion that these basidiospores were all produced by the *MAT*α *ric8* NAT-resistant strain (i.e. SSI867) through unisexual reproduction.

Next the KN99α *ric8* mutants (SSI867 and SSI868) were crossed with strain KN99**a** that is congenic with KN99α, and *MAT***a** *ric8*Δ::*NAT* progeny (SSI869 and SSI870, Supplemental Table S1) were recovered. Similar to strains SSI867 and SSI868, solo cultures of SSI869 and SSI870 underwent sexual development under mating inducing conditions (Figure 1). Analyses of four individual basidia produced by SSI869 revealed high germination rates in the basidiospores and showed that they were all *MAT***a** *ric8*Δ::*NAT*, again consistent with production via unisexual reproduction of the *MAT***a** *ric8* strain SSI869.

Taken together, these data indicate that deletion of the *RIC8* gene in the *C. neoformans* lab strains KN99**a** and KN99α enables unisexual reproduction, consistent with our previous findings in the sister species *C. deneoformans* (15).

### Naturally occurring variation promotes unisexual reproduction in *C. neoformans*

While the *C. neoformans* lab strains with a *ric8*Δ::*NAT* deletion are able to undergo unisexual reproduction, it occurs at relatively low frequencies and often requires prolonged incubation (≥ 4 weeks) under mating inducing conditions. We hypothesized that polymorphisms existing in natural isolates might promote unisexual reproduction. To test this, strain SSI867 (KN99α *ric8*Δ::*NAT*) was crossed with two *MAT***a** natural isolates (Bt63 and Bt65); strain SSI869 (KN99**a** *ric8*Δ::*NAT*) was crossed with two *MAT*α natural isolates (AD-17a and T4) (Table 2). A total of 56 random spores were dissected from each of these four crosses, with germination rates ranging from 18% (SSI867 × Bt65) to 34% (SSI869 × AD1-7a) (Table 2). Indeed, with the exception of the cross between SSI869 and T4, progeny were recovered with comparable or enhanced unisexual fertility compared to the unisexual parent (i.e. SSI867 or SSI869) in the other three crosses, at a percentage between 10% (3 out of 30 progeny, SSI867 × Bt63) and 30% (3 out of 10 progeny, SSI867 × Bt65) (Table 2). Interestingly, all of the unisexually fertile progeny inherited the *ric8*Δ::*NAT* allele from their selfing parent, further supporting that the ability to undergo unisexual reproduction requires the *RIC8* mutation (Table 2).

Of the unisexually fertile progeny that were recovered, one (SSK110) from the SSI869 × AD1-7a cross showed considerably enhanced selfing ability (Figure 1A and Table 2). SSK110 is a haploid *MAT*α strain (Figure 1B), with a genome that is colinear with the *C. neoformans* reference strain H99 (Figure 1C). Strain SSK110 was further crossed with natural isolate Bt63 (VNBI, *MAT***a**) and 70 random spores were dissected. Of the 29 progeny that germinated, five were able to self, with one progeny (SSK910) undergoing very robust unisexual reproduction under mating inducing conditions, producing extensive hyphae with unfused clamp cells and abundant basidia and basidiospore chains (Figure 1A). Strain SSK910 is a haploid *MAT***a** strain (Figure 1B), and its genome is nearly colinear with the reference strain H99, except for the reciprocal translocation involving chromosomes 3 and 11 that has been previously reported in the H99 genome (Figure 1C).

Self-fertile progeny from cross between SSK110 and Bt63 was further induced to undergo unisexual reproduction and random basidiospores were dissected. Whole genome sequencing and SNP analyses with H99 as reference showed that both the self-fertile parental strain and its progeny have identical genomes (Figure 1D), which are recombinants of alleles from KN99 (regions with no SNPs) and natural isolates (i.e. AD1-7a and Bt63, regions in blue indicating variants against H99) across the genome (Figure 1D).

In summary, through intercrossing with natural isolates, we successfully generated *C. neoformans* strains that undergo robust unisexual reproduction, confirming the presence of natural variants that promote selfing.

### Unisexual reproduction in *C. neoformans* requires a functional MAPK pathway

With the *C. neoformans* strain SSK910 that undergoes robust unisexual reproduction, we sought to investigate the signaling pathways required for this process. We constructed deletion strains for 14 genes that have been previously shown to be involved in mating in *C. neoformans* in the SSK910 background, including those involved in G-protein signaling, mating pheromone sensing, the MAP kinase pathway, sexual development, and meiosis (Table 3). We then studied whether these deletion strains were impaired for unisexual reproduction, including hyphal growth, basidium formation, and basidiospore production.

**Figure 2.**
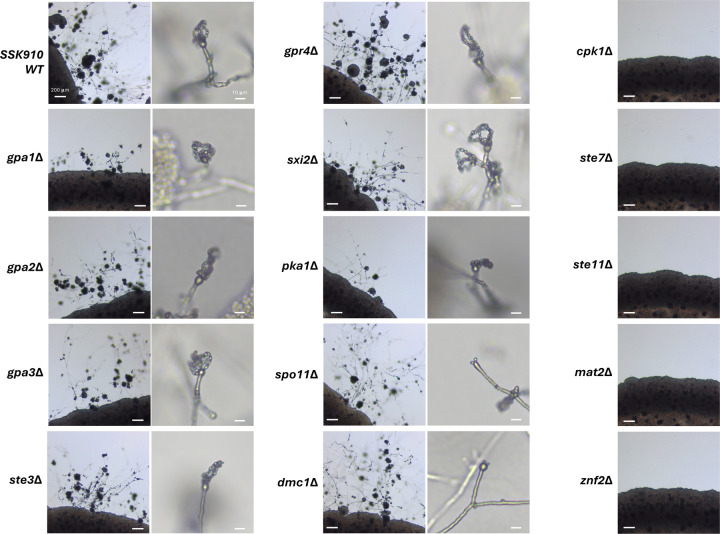
Unisexual reproduction in *C. neoformans* is dependent upon the MAPK pathway. Light microscopy images of strains with deletions of various genes in the SSK910 background when induced for unisexual reproduction on MS medium. Images shown here are from one of at least two independent deletion strains of each gene. Images on the right showed the basidia with or without basidiospore chains for the deletion strains that were still able to undergo unisexual reproduction.

Among the 14 genes tested, seven were not required for unisexual reproduction, and they are involved in G-protein signaling (*GPA1*, *GPA2*, *GPA3*), PKA signaling (*PKA1*), mating pheromone or nutrient sensing (*STE3* and *GPR4*), and the α-**a** sexual reproduction specific transcription factor (*SXI2*) (Figure 2 and Table 3) (27, 29–31). It should be noted that the deletions of *GPA1* and *PKA1* resulted in noticeably delayed unisexual reproduction, although in these cases, hyphae, basidia, and basidiospore chains were eventually produced within the selfing patches. Five genes were essential for unisexual reproduction, as deletion of these genes led to complete abolition of selfing, and the deletion strains grew only as yeast cells under mating inducing conditions (Figure 2). Three of these five genes (*CPK1*, *STE7*, and *STE11***a**) are components of the MAP kinase signaling pathway (32), while the other two (*MAT2* and *ZNF2*) are known master regulator transcription factors for α-**a** sexual reproduction in *C. neoformans* and unisexual reproduction in *C. deneoformans* (33). Additionally, while strains deleted for *SPO11* or *DMC1*, two genes known to be required for meiosis (34, 35), were able to undergo the initial stages of unisexual development, producing hyphae and basidia, the production of basidiospore chains was severely impaired, consistent with compromised meiosis in these strains (Figure 2).

Taken together, these results show that while the genes involved in G-protein signaling and mating pheromone sensing are dispensable, successful unisexual reproduction in *C. neoformans* requires a functional MAP kinase pathway, the master regulators of sexual reproduction, and genes essential for meiosis.

### Meiotic recombination occurs during unisexual reproduction in *C. neoformans*

Our results from gene deletion analyses shows that unisexual reproduction in *C. neoformans* involves genes required for meiosis. We next investigated whether unisexual reproduction in *C. neoformans* involves canonical meiotic recombination and if so, how this compares to α-**a** sexual reproduction. To accomplish this, one approach could be to analyze progeny from crosses between two genetically divergent strains of the same mating type. However, this approach is not feasible for a couple of reasons. First, sexual reproduction between two *C. neoformans* wild-type isolates of the same mating type has not yet been observed and may occur at a low frequency due to bottlenecks such as cell-cell fusion. Second, it is possible that sexual reproduction could occur between two divergent *C. neoformans ric8*Δ::*NAT* isolates that each could undergo unisexual reproduction. However, in this case it would not be possible to differentiate morphologically between spores produced by selfing of each parent strain and those generated by crossing between the two parental isolates, thus complicating the analyses of meiotic products, because those from selfing lack genetic polymorphisms and thus are not suitable for analyzing meiotic recombination.

**Figure 3.**
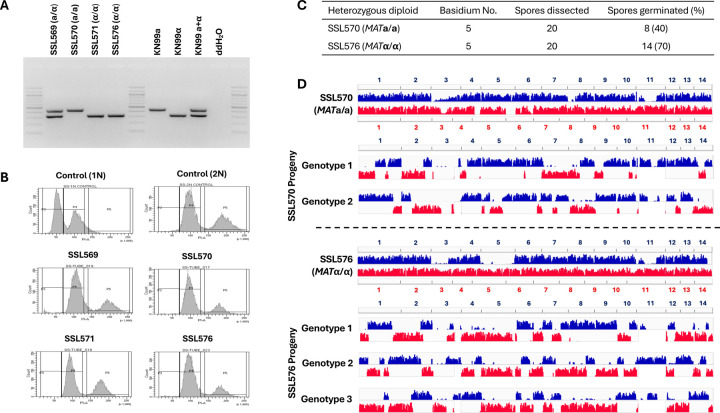
Unisexual reproduction in *C. neoformans* involves meiotic recombination. (A) Genotyping of the *MAT* locus showed that the three derived diploid strains, SSL570, SSL571, and SSL576, all underwent LOH at the mating type locus. (B) FACS analysis showed that the three derived diploid strains all have a diploid ploidy profile. (C) Summary of two basidia dissected from strains SSL570 and SSL576, respectively. (D) Variant mapping of the parental strains and their respective basidiospores. For the parental strains SSL570 and SSL576, the variants against H99 (blue) and Bt63 (red) were mapped along the H99 and Bt63 genomes, respectively. For the progeny, the variants against H99 and Bt63 were all mapped with the H99 genome as reference.

To overcome this, we first generated diploid *MAT***a**/**a** and *MAT*α/α strains that are homozygous for the *ric8*Δ/*ric8*Δ deletion (enabling selfing) and also contain heterozygous polymorphisms throughout the genome (allowing analyses of allele segregation). Specifically, we generated a *ric8*Δ::*NEO* deletion strain in the natural isolate Bt63 background (SSL436). Next, we fused strain SSL436 (*MAT***a** *ric8*Δ::*NEO*) with SSK110 (*MAT*α *ric8*Δ::*NAT*), a recombinant progeny from cross between lab strain KN99**a** *ric8*Δ::*NAT* and natural isolate AD1-7a, and recovered fusion products that were resistant to both *NAT* and *NEO*, and then screened for those that also exhibited reduced self-fertile sexual reproduction (<1%) when compared to *C. neoformans MAT***a** × *MAT*α sexual mating controls under mating inducing conditions, indicative of loss of heterozygosity at the *MAT* locus resulting in *MAT***a**/**a** or *MAT*α/α strains undergoing unisexual reproduction. By this approach, we successfully isolated three diploid fusion products between strains SSK110 and SSL436 that are *MAT*-homozygous and genome-heterozygous: SSL570 (*MAT***a**/**a**), SSL571 (*MAT*α/α), and SSL576 (*MAT*α/α) (Figure 3A, Table S2). FACS analyses confirmed that these fusion products are diploid (Figure 3B) and whole genome sequencing and variant analyses further showed that these strains contain heterozygosity throughout their genomes (Supplemental Figure S1, variants against H99 genome indicated in blue and variants against the Bt63 genome highlighted in red).

We then induced unisexual reproduction in diploid strains SSL570, SSL571, and SSL576 under mating inducing conditions, and dissected spores from individual basidia (Table 1) from each strain. Compared to the basidiospores dissected from unisexual reproduction of haploid *ric8*Δ strains (e.g. SSI876 and SSK910, Table 1), the germination rates of spores from selfing of the diploid *MAT*-homozygous strains were considerably lower, which is reflective of both the genetic diversity and one chromosomal translocation between the two nuclear genomes in the diploids. This is consistent with the reduced spore germination rates that have been observed previously in crosses between *C. neoformans* lab strains and natural isolates. It should be noted that all of the progeny share the same mating type as their parental diploid strains, further confirming that these progeny are products of unisexual reproduction (Table 1).

Whole-genome-sequencing and variant analyses of the progeny dissected from these *MAT*-homozygous diploids shows clear evidence of allele segregation consistent with meiotic recombination (Figure 3D, Supplemental Figures S2 – S4). For example, of the 20 spores dissected from basidium No. 5 of strain SSL570 (*MAT***a**/**a**), eight germinated, which is a 40% germination rate (Figure 3C). Because in *C. neoformans* only one round of meiosis occurs within each basidium, giving rise to four recombinant meiotic products that undergo repeated rounds of mitosis to produce basidiospores (36), we would expect two distinct nuclear genotypes among these eight germinated spores given the ~50% germination rate, assuming the other two genotypes failed to germinate due to the factors such as the known chromosomal translocations, as well as the segregation of other genetic polymorphisms between the two nuclear genomes. Indeed, we observed two types of progeny among these eight spores, each having recombinant genomes with alleles from the two parental nuclei alternating along each chromosome, consistent with products of meiotic crossovers (Figure 3D, top panel). Similarly, 14 of the 20 spores dissected from strain SSL576 basidium No. 5 germinated, and the 70% germination rate suggested that three of the four meiotic products gave rise to viable basidiospores. Indeed, we observed three different genotypes among these progeny (Figure 3D, bottom panel). Similar to those from SSL570 basidium No. 5, these progeny also possessed recombinant chromosomes that were consistent with meiotic crossovers. Additionally, no allele from either of the two parental genomes was observed in more than two progeny genotypes, providing further support that these progeny are products of meiosis.

Collectively, these analyses provided robust evidence that meiotic recombination occurs during unisexual reproduction in *C. neoformans*.

### Ric8 is involved in localization of Gpa1 and Gpa2 during α-a sexual reproduction but not unisexual reproduction

**Figure 4.**
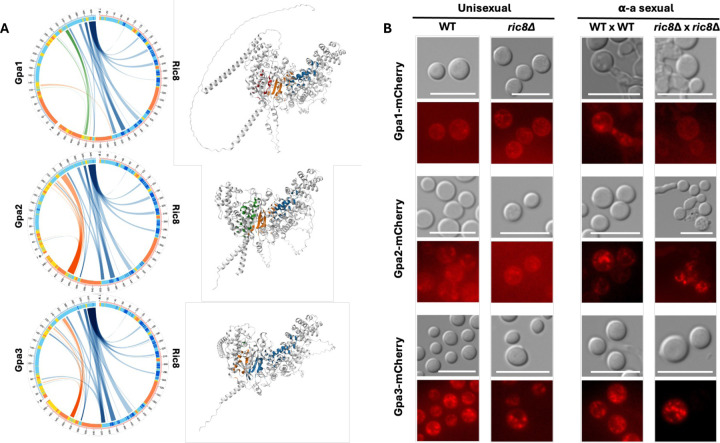
Interactions between Ric8 and the three Gα proteins in *C. neoformans*. (A) AlphaFold predictions of the 3D structures and protein-protein interaction profiles between Ric8 and the three Gα proteins (Gpa1, Gpa2, and Gpa3). For each Ric8-Gα protein pair, the circus plot on the left illustrates the residues predicted to be interacting between the two proteins, and the image on the right showes the predicted 3D structure of the protein complex, colored for the interfaces. (B) Subcellular localization of endogenously mCherry-tagged Gα proteins during unisexual reproduction (left) and α-a sexual reproduction (right) in *C. neoformans*. The mCherry-tagged strains are in either the KN99α wild-type or KN99α *ric8*Δ::*NAT* strain background. For unisexual reproduction, individual strains were spotted on MS medium as solo-cultures (left panels). For α-a sexual reproduction, each *MAT*α mCherry-tagged strain (wild-type or *ric8*Δ) was mated with a *MAT*a strain of the same *RIC8* genotype on MS medium. Cells were imaged after 5 days of incubation under mating inducing conditions. Shown here are differential interference contrast (DIC, top) and mCherry fluorescence (bottom) images. Scale bar=10 μm.

Of the three Gα proteins in *C. neoformans*, Ric8 has been previously shown to interact with Gpa1 and Gpa2, but not Gpa3, based on yeast two hybrid assays and co-immunoprecipitation analysis (27). Interestingly, when we predicted protein-protein interactions between Ric8 and the three Gα proteins using AlphaFold multimer, our results suggest that Ric8 might form complexes with each Gα protein with similar interaction profiles of comparable confidence (Figure 4A).

To further investigate the interactions between Ric8 and the three Gα subunits during mating, we examined the subcellular localizations of Gpa1, Gpa2, and Gpa3 under mating-inducing conditions. Specifically, we first constructed strains with mCherry-tagged Gα proteins in both the KN99α wild-type (WT) and KN99α *ric8*Δ::*NAT* mutant backgrounds. Then for unisexual reproduction, we spotted each of the *MAT*α strains as solo cultures on MS medium and incubated them under mating inducing conditions. We observed no noticeable differences between the localization of Gα proteins between the WT and the *ric8*Δ strains, although the mCherry signals formed more pronounced puncta for Gpa3 compared to Gpa1 or Gpa2 (Figure 4B).

For α-**a** sexual reproduction, we crossed each of the mCherry-tagged *MAT*α strains with a *MAT***a** strain of the same *RIC8* genotype (i.e. WT × WT and *ric8*Δ × *ric8*Δ) and incubated the cultures under mating inducing conditions. Consistent with our previous observations, the *ric8*Δ × *ric8*Δ crosses showed reduced mating compared to the crosses between *RIC8* wild-type strains, suggesting the mCherry tagging of the Gα proteins did not introduce phenotypic changes during mating. After 5 days of incubation, we observed similar localization patterns for the three Gα proteins in the WT × WT crosses, as all three proteins formed aggregated puncta in the cytoplasm (Figure 4B). However, in the *ric8*Δ × *ric8*Δ crosses, while the localization of Gpa3 is similar to that in the WT × WT cross, the localizations of Gpa1 and Gpa2 appear different. Specifically, the mCherry signal for Gpa1 was markedly reduced and appeared to be diffused in the cytoplasm, while Gpa2 exhibited a broader distribution in the cytoplasm without any clear compartmentalization, suggesting that Ric8 may contribute to the spatial regulation of Gpa1 and Gpa2 during α-**a** sexual reproduction (Figure 4B).

Thus, our results suggest that Ric8 is selectively required for the proper localization or stability of specific Gα proteins, particularly Gpa1 and Gpa2, during α-**a** sexual reproduction but not unisexual reproduction.

## Discussion

We have discovered that unisexual reproduction can occur in *C. neoformans*, the predominant global pathogenic form of this human fungal pathogen. Our findings further demonstrate that the human fungal pathogen *Cryptococcus* species complex exhibits great plasticity in the ability to achieve successful sexual reproduction, including α-**a** sexual reproduction, unisexual reproduction, and pseudosexual reproduction (5, 8–10, 13). All three modes of sexual reproduction involve hyphal growth, meiosis, and production of basidiospores, which allow the organism to “forage” for nutrients or mating partners over long distances, generate recombinant progeny with potentially higher fitness, and produce basidiospores that are more resilient under challenging conditions and better equipped for dispersal.

We showed that there are variants in the natural population of *C. neoformans* that promote unisexual reproduction. Specifically, while the initial *ric8*Δ mutation in the lab strain KN99**a** and KN99α backgrounds only led to sporadic unisexual reproduction, strains SSK110 and SSK910, which are progeny from intercrosses with natural isolates, exhibited significantly enhanced selfing ability. This suggests that it is possible that there are natural strains with enhanced ability to undergo unisexual reproduction. The natural population of *Cryptococcus* species exhibits significant bias towards the α mating type, with populations of balanced mating types identified only in certain geographic areas (e.g. Sub-Saharan Africa) or certain lineages (e.g. VNBI and VNBII) (11). Thus, the enhanced ability to undergo unisexual reproduction could be favored by natural selection as it allows *Cryptococcus* to undergo sexual development that is beneficial under challenging conditions even in the absence of cells of the opposite mating types required for α-**a** sexual reproduction, and might have emerged as *C. neoformans* became globalized and went through genetic bottlenecks resulting in populations with severely imbalanced mating types.

Our findings also highlight the importance of studying strains with diverse genetic backgrounds, including natural strains, to acquire a more complete understanding of biological processes. While studying lab reference strains has its advantages, these isolates could have accumulated private mutations that make them unique compared to other isolates in the species. For example, a recent study suggested that the *C. neoformans* reference strain, H99, possesses a unique mutation in the gene *PDE2* that might be contributing to its elevated virulence in the host (37). Another recent study showed that in *C. neoformans* dysfunction in the RNAi pathway leads to hypermutation when combined with and elevated transposon burden in the genome, which was also discovered in natural isolates as the lab strain H99 has a genome with a very low transposon burden (38). Furthermore, the human fungal pathogen *Candida albicans* had been long thought to be RNAi deficient based on the analyses of the reference strains, SC5314, as it contains an inactivating missense mutation in the gene encoding Argonaute, a central RNAi component. However, a recent study of natural *C. albicans* strains showed that the mutation in Argonaute is rare, and the species contains an intact and functional RNAi pathway (39). Thus, what we learn from reference strains may not be representative for the species as a whole.

Our study provides further evidence that *RIC8* is a key regulator of morphological development and a suppressor of unisexual reproduction in *Cryptococcus* species. This is in accord with our previous study in *C. deneoformans* where the presence of a functional *RIC8* restricted the strains to grow as yeast cells under mating inducing conditions, whereas deletion of the *RIC8* gene enabled cells to undergo morphological changes, including hyphal growth and unsexual reproduction, as well as an elevated ability of titan cell formation (15). In *C. deneoformans*, where unisexual reproduction was first characterized, the strain that undergoes the most robust unisexual reproduction, XL280, also contains a nonsense mutation in the *RIC8* gene (15). Additionally, during our effort to construct *C. neoformans* strains that undergo robust unisexual reproduction, all of the selfing progeny inherited the *ric8*Δ deletion allele. Thus, *RIC8* appears to be a critical regulator of morphological development in *Cryptococcus* species. The *RIC8* gene encodes a guanine nucleotide exchange factor (GEF) for the Gα subunit of heterotrimeric G proteins, and our analyses indicate that while it structurally may form protein complexes with all three Gα proteins similarly, functionally the deletion of Ric8 only affected the localization of Gpa1 and Gpa2 during α-**a** sexual reproduction but not during unisexual reproduction. Ric8 could function through modulating G-protein coupled receptor (GPCR) signaling pathways, by facilitating GDP-to-GTP exchange and activating Gα subunits, as well as serving as a chaperone for the Gα proteins, which in turn interact with downstream effectors and initiate various intracellular signaling pathways (40, 41). It is possible that under mating inducing conditions, Ric8 activates Gα proteins (Gpa1 and Gpa2) that favors α-**a** sexual reproduction and represses unisexual reproduction, which is also consistent with the observation that *ric8*Δ strains exhibit significantly reduced mating in α-**a** sexual reproduction (15). In contrast, the absence of Ric8 would enable cells to initiate sexual reproduction without the presence of compatible mating types when conditions become favorable for mating, which could be through modulation of cell ploidy (haploid to diploid) and/or morphology (yeast to hyphae). Further studies should aim to elucidate how the switching between α-**a** sexual reproduction and unisexual reproduction is regulated by the *RIC8* gene, as well as whether this regulatory pathway is conserved in other fungal species.

Different modes of sexual reproduction in *Cryptococcus* involve shared molecular pathways, including a functional MAPK pathway for signal transduction; transcription factors that play central roles in regulating morphological development during sexual reproduction (e.g. Mat2 and Znf2); and key factors such as Dmc1 and Spo11 for meiosis and sporulation. Thus, the difference mainly lies in how sexual reproduction is initiated. It is known that certain environmental cues, including certain nutrients and temperature, induce sexual reproduction in *Cryptococcus*. Additionally, sexual reproduction in *Cryptococcus* may also be a response to biotic factors in the environment, such as fungal/bacterial cohabitants and their secondary metabolites, which can induce stress response, lead to transcriptome fluctuation, and modulate morphological changes, and for which many of the response pathways, including MAPK, cAMP-PKA, calcineurin, and TOR pathways, have been shown to be involved in regulating morphogenesis and sexual development in *Cryptococcus* (42).

As unicellular eukaryotes, *Cryptococcus* is sensitive to environmental changes. Being able to initiate diverse modes of sexual reproduction, α-**a** sexual reproduction when both mating types are present and unisexual reproduction when only one mating type is available, allows *Cryptococcus* to grow by mitotic propagation under favorable environmental conditions, and rapidly undergo sexual reproduction when challenging environmental conditions arise. It is possible that the Ric8-mediated regulatory pathway for unisexual reproduction is conserved in other fungal species, and might be modulated by environmental factors that remain to be characterized. Unisexual reproduction may occur in other fungi, shaping the ecology, population dynamics, and evolutionary trajectory of not only the pathogenic *Cryptococcus* species, but also other fungal species and beyond.

## Materials and methods

### Strain handling and culturing

All of the strains were grown on YPD medium, supplemented with appropriate antifungal drugs (nourseothricin (NAT) at 100 μg/mL or neomycin (G418) at 200 μg/mL) when needed, and incubated at 30 °C unless stated otherwise.

### DNA preparation and whole genome sequencing

DNA for genotyping and Illumina whole genome sequencing were prepared using a MasterPure Yeast DNA Purification Kit (LGC Biosearch Technologies, MPY80200) as previously described (43). Illumina sequencing was conducted at the Duke Sequencing and Genomic Technologies core facility (https://genome.duke.edu), on a Novaseq 6000 platform with 250 bp paired-end option.

High molecular weight genomic DNA samples for Nanopore long read sequencing were prepared using a modified CTAB protocol as previously described (5). Nanopore long-read sequencing was conducted in house using a MinION device with R10.4.1 flow cells following instructions provided by the manufacturer.

All of the sequencing data have been deposited in the BioProject ID PRJNA1269048.

### Deletion strain construction

Gene deletions using the *NAT* or *NEO* dominant markers were conducted using a TRACE CRISPR system, and transformants were validated by genotyping using diagnostic PCRs for the internal region of the ORF, 5’-junction and 3’-junction regions, as well as region encompassing the deletion construct as previously described (44, 45).

### Mating and spore dissection

The mating assay and spore dissection were carried as described in previously published protocols (46). Briefly, all of the mating assays were conducted on MS solid medium at room temperature in the dark. Plates were inspected for sexual development after 1–2 weeks of incubation and kept for prolonged incubation when needed. Basidiospore dissection was carried out using a Nikon yeast spore dissection microscope equipped with a glass fiber needle 25 μm in diameter.

### Flow cytometry analysis

Samples for fluorescence-activated cell sorting (FACS) analysis to determine Cryptococcus ploidy was prepared as previously described (43, 47). The FACS analysis was performed at the Duke Cancer Institute Flow Cytometry Shared Resource Laboratory.

### Microscopy imaging

Brightfield and differential interference contrast (DIC) microscopy images were acquired with an AxioScop 2 fluorescence microscope equipped with an AxioCam MRm digital camera (Zeiss, Germany). Scanning electron microscopy (SEM) was performed at the Duke Microscopy Core Facility using a scanning electron microscope with an EDS detector (Apreo S, ThermoFisher, USA).

## Supplementary Material

Supplement 1 Supplemental Figure S1. Genotypic profiles of the unisexually fertile *C. neoformans* strains.
(A) Genotyping of the *MAT* locus for the haploid strains analyzed in this study. (B) Genome analysis of the strains that unisexually reproduce. The blue and red colors indicate SNPs when mapped against the H99 and Bt63 genomes, respectively. For each strain, zoomed-in read coverage maps of chromosome 5 are shown with. the H99 (blue) or Bt63 (red) genome as reference. For strains SSK110 and SSK910, additional zoomed-in maps of SNPs against H99 (blue) or Bt63 (red) along chromosome 5 are included beneath the read-depth plots.

Supplement 2 Supplemental Figure S2. Analyses of progeny from diploid *MAT*a/a *C. neoformans* strain SSL570.
Variant mapping of the parental strain SSL570 and its dissected basidiospore chains. For the parental strain SSL570, the variants against H99 (blue) and Bt63 (red) were mapped against the H99 and Bt63 genomes, respectively. For the progeny, the variants against H99 and Bt63 were all mapped with the H99 genome as reference. The purple column indicates the location of the *MAT* locus.

Supplement 3 Supplemental Figure S3. Analyses of progeny from diploid *MAT*α/α *C. neoformans* strain SSL571.
Variant mapping of the parental strain SSL571 and its dissected basidiospore chains. For the parental strain SSL571, the variants against H99 (blue) and Bt63 (red) were mapped against the H99 and Bt63 genomes, respectively. For the progeny, the variants against H99 and Bt63 were all mapped with the H99 genome as reference. The purple column indicates the location of the *MAT* locus.

Supplement 4 Supplemental Figure S4. Analyses of progeny from diploid *MAT*α/α *C. neoformans* strain SSL576.
Variant mapping of the parental strain SSL576 and its dissected basidiospore chains. For the parental strain SSL576, the variants against H99 (blue) and Bt63 (red) were mapped against the H99 and Bt63 genomes, respectively. For the progeny, the variants against H99 and Bt63 were all mapped with the H99 genome as reference. The purple column indicates the location of the *MAT* locus.

Supplement 5 Supplemental Figure S5. Interaction between Ric8 and the three Ga proteins in *C. neoformans*
(A) Plots of the Predicted Aligned Error (PAE) for the protein complexes between Ric8 and each of the three Ga proteins (Gpa1, Gpa2, and Gpa3; top to bottom) generated by AlphaFold multimer prediction. Color coding from green to white indicates low to high PAE values, corresponding to gradually decreased confidence for the structural predictions. (B) Subcellular localization of endogenously mCherry-tagged Ga proteins during unisexual reproduction (left) and α-**a** sexual reproduction (right) in *C. neoformans*. The mCherry-tagged strains are in either the KN99α wild-type or KN99α *ric8*Δ::*NAT* strain background. For unisexual reproduction, individual strains were spotted on MS medium as solo-cultures (left panels). For α-**a** sexual reproduction, each *MAT*α mCherry-tagged strain (wild-type or *ric8*Δ) was mated with a *MAT***a** strain of the same *RIC8* genotype on MS medium. Cells were imaged after 5 days of incubation under mating inducing conditions. Shown here are differential interference contrast (DIC, top) and mCherry fluorescence (bottom) images. Scale bar=10 μm.

6

## Figures and Tables

**Table 1.** Unisexual reproduction and progeny sets generated and analyzed in this study.

Selfing	Parental strain genotype	Basidium No.	Spores dissected	Spores germinated	Spore mating type
SSI867	KN99α *ric8*Δ::*NAT*	1	13	11	All *MAT*α
		2	23	21	All *MAT*α
		3	12	9	All *MAT*α
		4	14	13	All *MAT*α
		5	30	23	All *MAT*α
SSI869	KN99**a** *ric8*Δ::*NAT*	1	20	17	All *MAT***a**
		2	18	17	All *MAT***a**
		3	12	10	All *MAT***a**
		4	17	16	All *MAT***a**
SSK910	Recombinant progeny from SSK110 × Bt63	1	7	6	All *MAT***a**
		2	6	5	All *MAT***a**
		3	14	13	All *MAT***a**
		4	20	20	All *MAT***a**
SSL570	Fusion (Bt63 *ric8*Δ::*NEO* + SSK110) *MAT***a**/**a**	1	10	4	All *MAT***a**
		2	12	0	n.a.
		3	5	2	All *MAT***a**
		4	14	4	All *MAT***a**
		5	20	8	All *MAT***a**
SSL571	Fusion (Bt63 *ric8*Δ::*NEO* + SSK110) *MAT*α/α	1	10	0	n.a.
		2	11	6	All *MAT*α
		3	11	0	n.a.
SSL576	Fusion (Bt63 *ric8*Δ::*NEO* + SSK110) *MAT*α/α	1 ^1^	28	17	All *MAT*α
		2	23	7	All *MAT*α
		3	17	2	All *MAT*α
		4	7	3	All *MAT*α
		5	20	14	All *MAT*α
		6	14	6	All *MAT*α
		7	12	3	All *MAT*α
		8	5	0	n.a.

1: This is likely a mixture of 2 or 3 basidia based on WGS and SNP analyses (Supplemental Figure S4)

**Table 2.** α-**a** sexual crosses and progeny sets generated and analyzed in this study.

Cross	Spores dissected	Spores germinated (%; progeny IDs)	Self-fertile^1^	Self-sterile
SSI867 (KN99α *ric8*Δ::*NAT*) × Bt63	56	30 (54%; K961 – K990)	3	27
SSI867 (KN99α *ric8*Δ::*NAT*) × Bt65	56	10 (18%; K099 – K108)	3	7
SSI869 (KN99**a** *ric8*Δ::*NAT*) × AD1-7a	56	19 (34%; K109 – K127)	2	17
SSI869 (KN99**a** *ric8*Δ::*NAT*) × T4	56	11 (20%; K128 – K138)	0	11
SSK110 × Bt63	70	29 (41%; K910 – K938)	5	24

1: All of the selfing progeny inherited the *ric8*Δ::*NAT* allele.

**Table 3.** Genes deleted in the self-fertile strain.

Gene ID ^1^	Gene Name	Encoded protein	Self-fertility	Sporulation
CNAG_04505	*GPA1*	Guanine nucleotide-binding protein subunit α	Slightly delayed	Yes
CNAG_00179	*GPA2*	G protein α subunit	Yes	Yes
CNAG_02090	*GPA3*	G protein α subunit	Yes	Yes
CNAG_06808	*STE3*	G-protein coupled receptor	Yes	Yes
CNAG_04730	*GPR4*	G-protein coupled receptor	Yes	Yes
n.a.	*SXI2*	Homeodomain (HD) transcription factor	Yes	Yes
CNAG_00396	*PKA1*	Protein kinase A	Slightly delayed	Yes
CNAG_05472	*SPO11*	Meiotic recombination protein	Yes	No
CNAG_07909	*DMC1*	Meiotic recombinase	Yes	No
CNAG_02511	*CPK1*	MAPK	No	n.a.
CNAG_01730	*STE7*	MAPKK	No	n.a.
CNAG_06980	*STE11*	MAPKKK	No	n.a.
CNAG_06203	*MAT2*	HMG-box transcription factor	No	n.a.
CNAG_03366	*ZNF2*	Master regulator of filamentation, C2H2 type zinc finger	No	n.a.

1: The gene IDs are based on the annotation of the *MAT*α H99 genome. *SXI*2**a** does not have a gene ID as it is a *MAT***a** specific gene.
