# Supplementary figures and images for: Unisexual reproduction in the global human fungal pathogen Cryptococcus neoformans
Source: bioRxiv. 2025 Sep 2:2025.06.02.657540. Originally published 2025 Jun 3. Preprint. [Version 3] doi: 10.1101/2025.06.02.657540 (PMC12157407; doi:10.1101/2025.06.02.657540)

Supplemental Figure S1

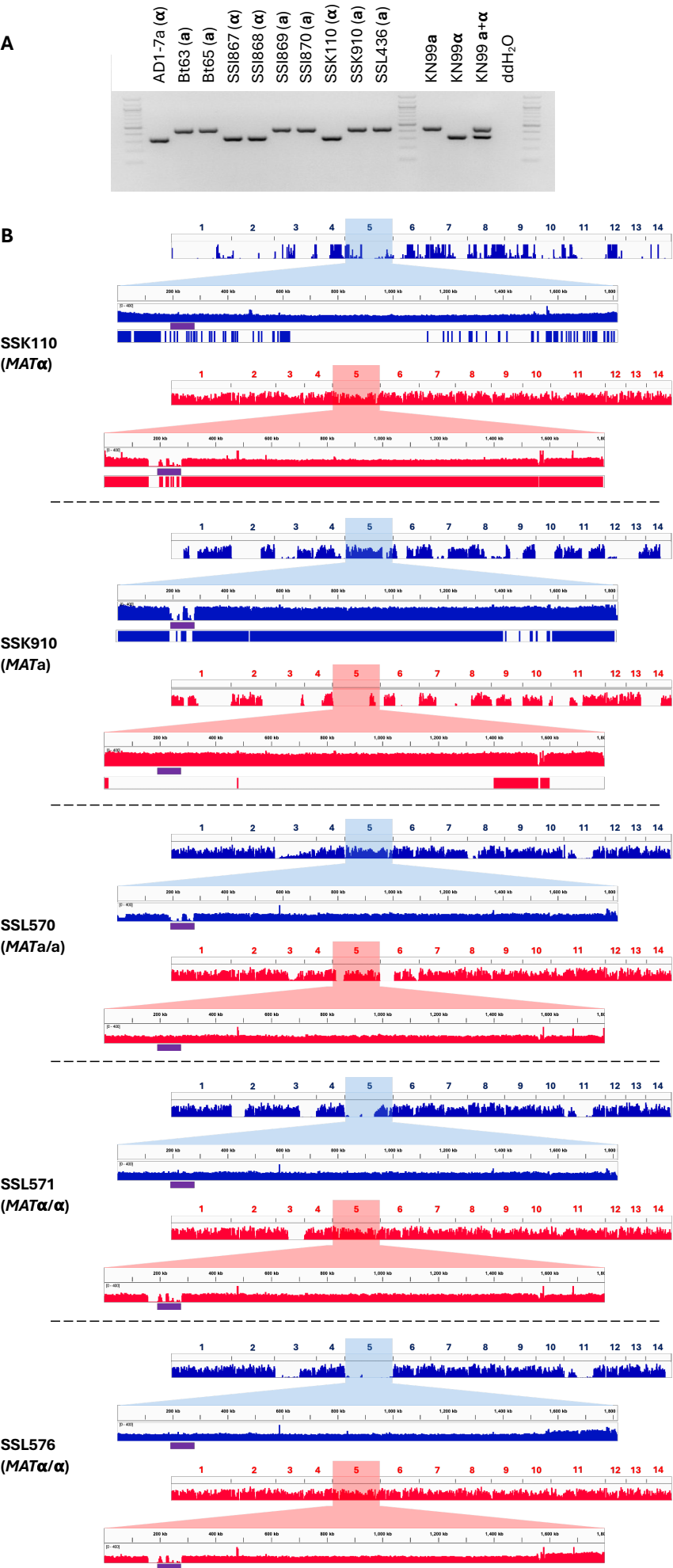

Supplement: Supplement 1 — Supplemental Figure S1. Genotypic profiles of the unisexually fertile C. neoformans strains. (A) Genotyping of the MAT locus for the haploid strains analyzed in this study. (B) Genome analysis of the strains that unisexually reproduce. The blue and red colors indicate SNPs when mapped against the H99 and Bt63 genomes, respectively. For each strain, zoomed-in read coverage maps of chromosome 5 are shown with. the H99 (blue) or Bt63 (red) genome as reference. For strains SSK110 and SSK910, additional zoomed-in maps of SNPs against H99 (blue) or Bt63 (red) along chromosome 5 are included beneath the read-depth plots. [file media-1.pdf]

Supplemental Figure S2

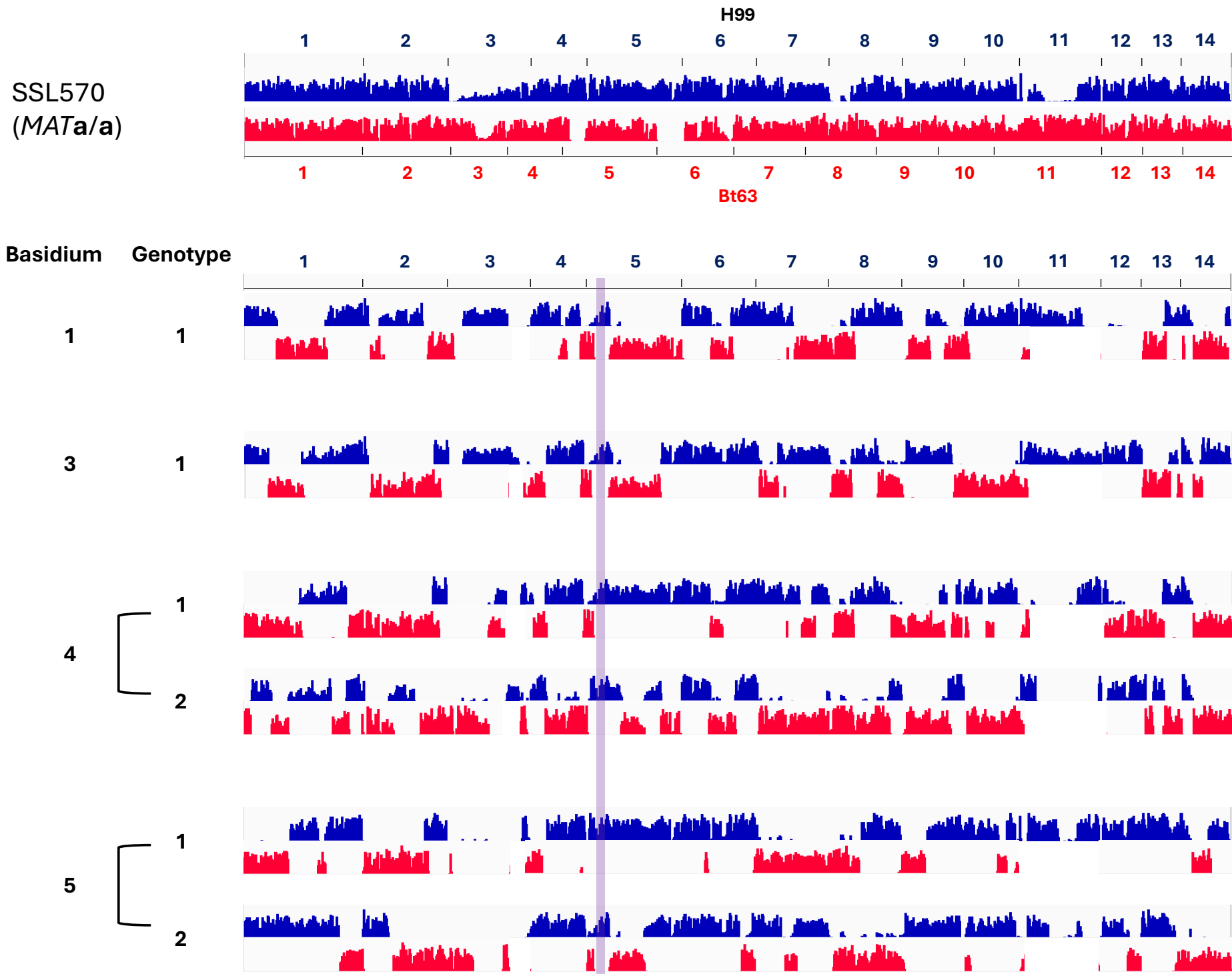

Supplement: Supplement 2 — Supplemental Figure S2. Analyses of progeny from diploid MATa/a C. neoformans strain SSL570. Variant mapping of the parental strain SSL570 and its dissected basidiospore chains. For the parental strain SSL570, the variants against H99 (blue) and Bt63 (red) were mapped against the H99 and Bt63 genomes, respectively. For the progeny, the variants against H99 and Bt63 were all mapped with the H99 genome as reference. The purple column indicates the location of the MAT locus. [file media-2.pdf]

Supplemental Figure S3

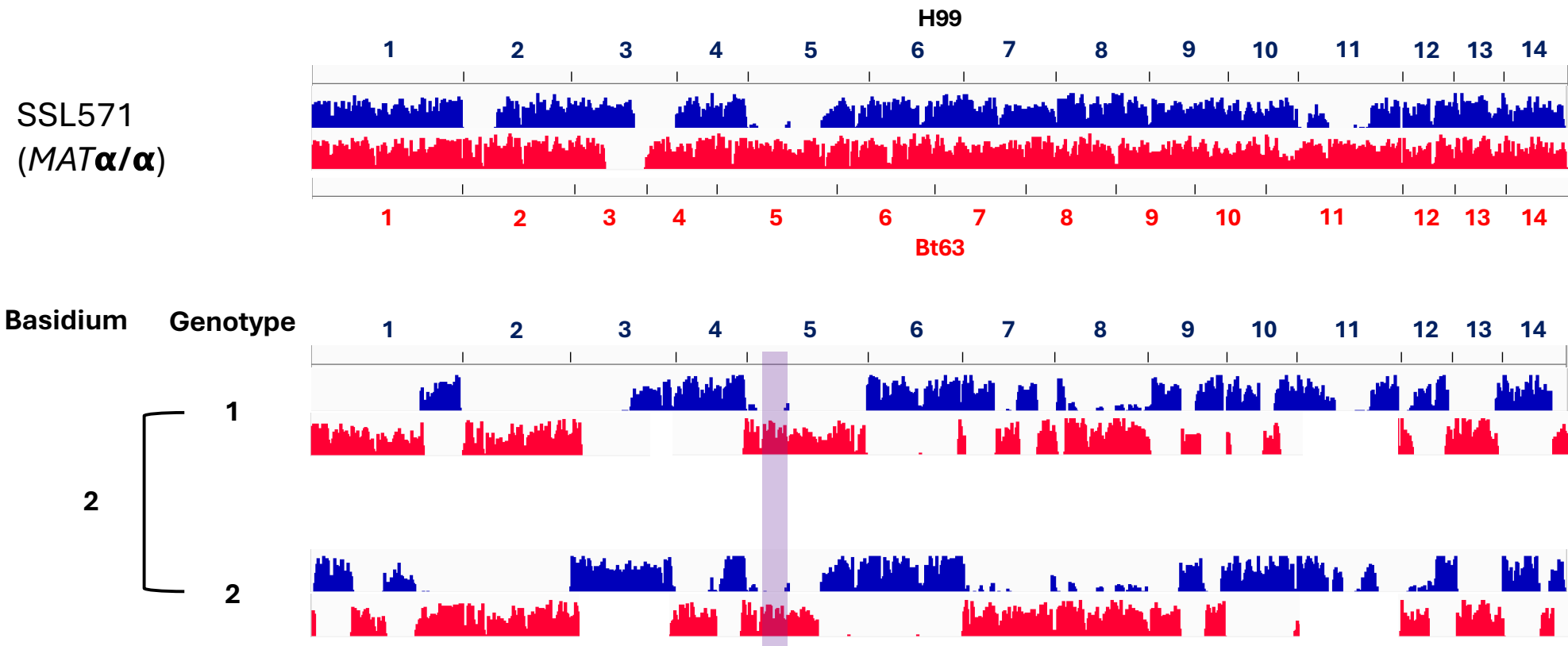

Supplement: Supplement 3 — Supplemental Figure S3. Analyses of progeny from diploid MATα/α C. neoformans strain SSL571. Variant mapping of the parental strain SSL571 and its dissected basidiospore chains. For the parental strain SSL571, the variants against H99 (blue) and Bt63 (red) were mapped against the H99 and Bt63 genomes, respectively. For the progeny, the variants against H99 and Bt63 were all mapped with the H99 genome as reference. The purple column indicates the location of the MAT locus. [file media-3.pdf]

### Supplemental Figure S4

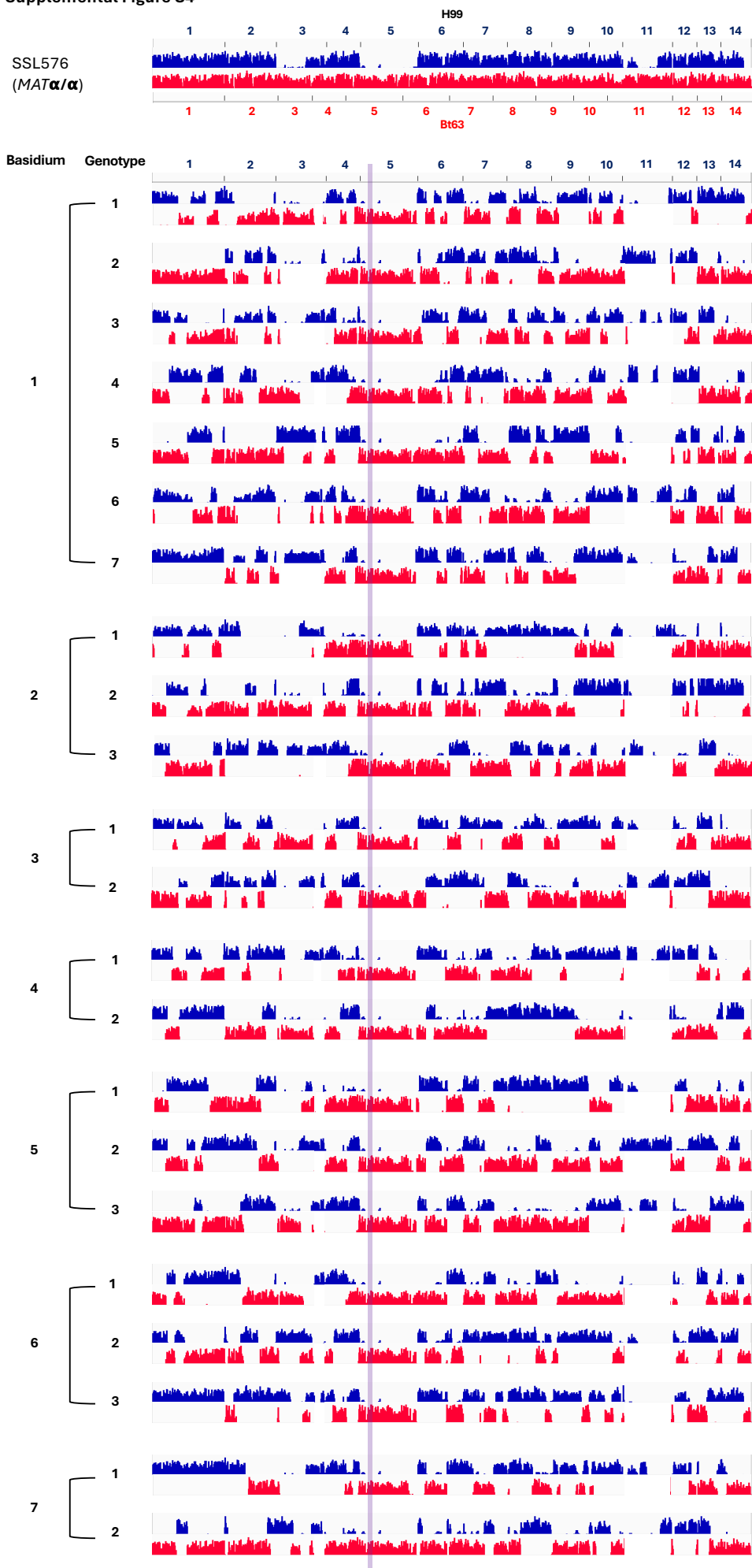

Supplement: Supplement 4 — Supplemental Figure S4. Analyses of progeny from diploid MATα/α C. neoformans strain SSL576. Variant mapping of the parental strain SSL576 and its dissected basidiospore chains. For the parental strain SSL576, the variants against H99 (blue) and Bt63 (red) were mapped against the H99 and Bt63 genomes, respectively. For the progeny, the variants against H99 and Bt63 were all mapped with the H99 genome as reference. The purple column indicates the location of the MAT locus. [file media-4.pdf]

### Supplemental Figure S5

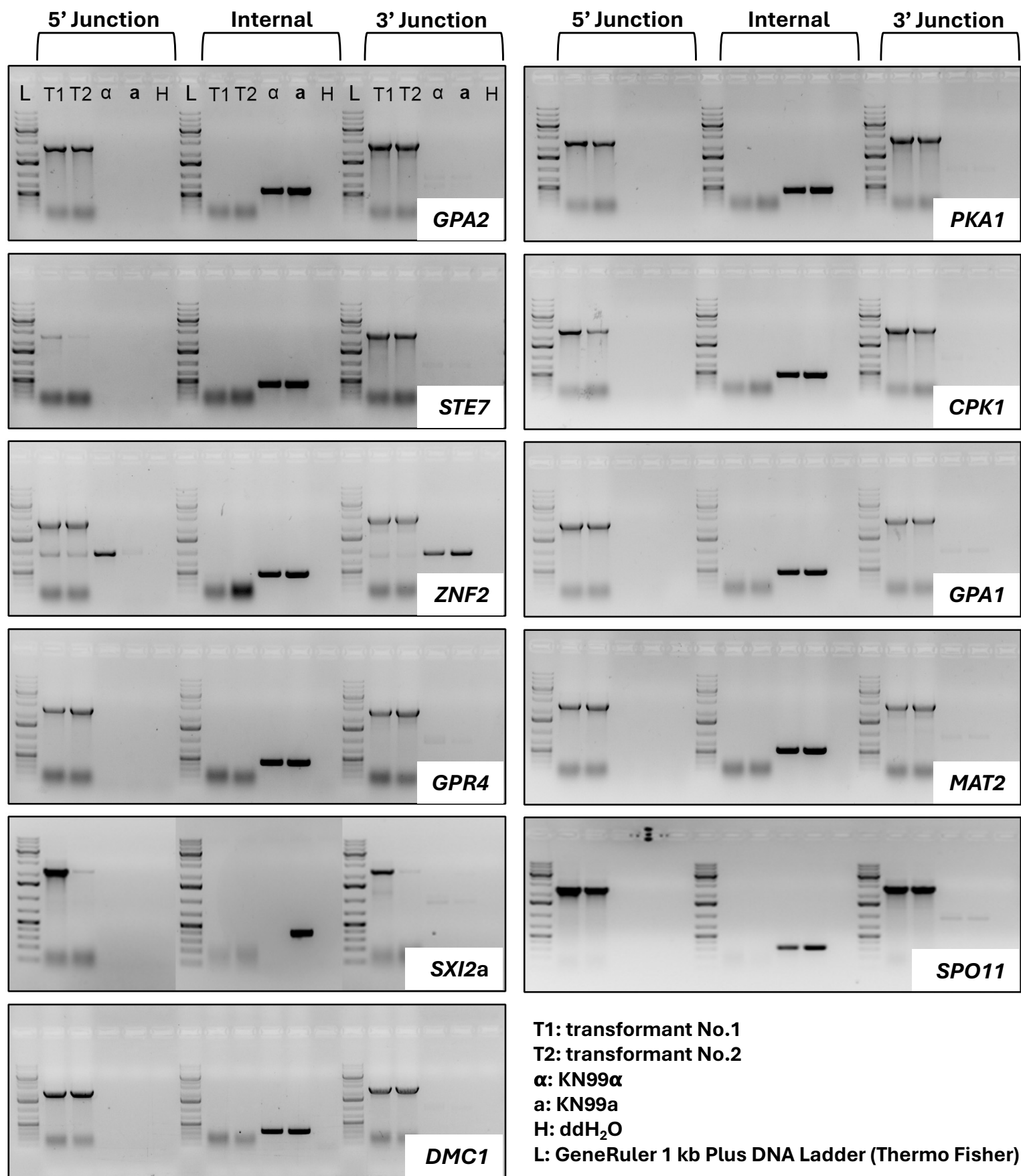

Supplement: Supplement 5 — Supplemental Figure S5. PCR validation of the deletion strains in the self-fertile SSK910 strain background. Each image demonstrates PCR validation of two independent deletion strains of one gene (labeled at the right bottom corner of the image), including 5’- and 3’- junction PCRs (left and right panels, respectively, amplifying only from deletion strains) and internal PCR (middle panel, amplifying only from wild-type controls). For each PCR reaction, samples from left to right are: deletion strain No.1, deletion strain No.2, KN99a wild-type control, KN99α wild-type control, and ddH2O negative PCR control. [file media-5.pdf]
