# Supplementary material for: Unisexual reproduction in the global human fungal pathogen Cryptococcus neoformans
Source: bioRxiv. 2025 Sep 2:2025.06.02.657540. Originally published 2025 Jun 3. Preprint. [Version 3] doi: 10.1101/2025.06.02.657540 (PMC12157407; doi:10.1101/2025.06.02.657540)

A

Strain Name	Mating Type	Genotype / Markers / Phenotype
YPH106	**a**	*gpa2***Δ***::NEO gpa3***Δ***::NEO*
YPH118	**a**	*crg1***Δ***::URA5 gpa2***Δ***::NEO gpa3***Δ***::NEO*
YPH305	α	*crg2***Δ***::NAT gpa2***Δ***::NEO gpa3***Δ***::NEO*
YPH308	α	*gpa2***Δ***::NAT gpa3***Δ***::NEO*
YPH380	α	*crg1***Δ***::URA5 gpa2***Δ***::NEO gpa3***Δ***::NEO*

B

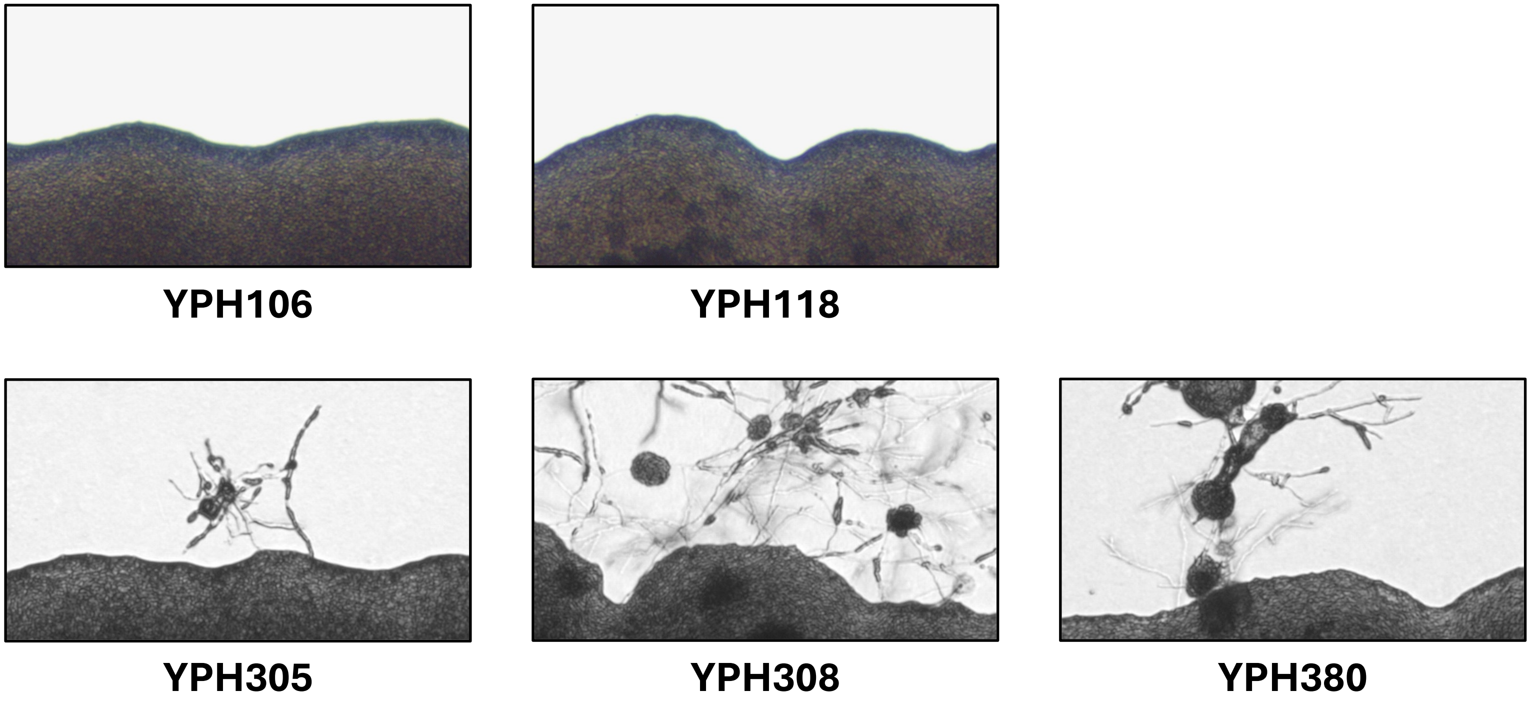

Supplement: Supplement 7 — Supplemental Figure S7. Selfing assay of gpa2Δ gpa3Δ mutant strains. (A) The five gpa2Δ gpa3Δ mutant strains constructed in a previous study (53). (B) Selfing assay of the five gpa2Δ gpa3Δ mutant strains under mating inducing condition. Images were taken after 10 days of incubation. [file media-7.pdf]
