# Supplementary material for: Unisexual reproduction in the global human fungal pathogen Cryptococcus neoformans
Source: bioRxiv. 2025 Sep 2:2025.06.02.657540. Originally published 2025 Jun 3. Preprint. [Version 3] doi: 10.1101/2025.06.02.657540 (PMC12157407; doi:10.1101/2025.06.02.657540)
Supplement: 1 [file NIHPP2025.06.02.657540V3-supplement-1.pdf]

Supplemental Table S1. Strains analyzed in this study.

Strain Name	Strain background	Genotype	Source
KN99α	n.a.	*MAT*α wildtype	(70)
KN99**a**	n.a.	*MAT***a** wildtype	(70)
AD1-7a	n.a.	*MAT*α wildtype	(19)
T4	n.a.	*MAT*α wildtype	(19)
Bt63	n.a.	*MAT***a** wildtype	(19)
Bt65	n.a.	*MAT***a** wildtype	(19)
SSI867	KN99α	*MAT*α *ric8*Δ::*NAT*	(27)
SSI868	KN99α	*MAT*α *ric8*Δ::*NAT*	(27)
SSI869	Progeny from SSI867 × KN99**a**	*MAT***a** *ric8*Δ::*NAT*	(27)
SSI870	Progeny from SSI868 × KN99**a**	*MAT***a** *ric8*Δ::*NAT*	(27)
SSK110	Progeny from SSI869 × AD1-7a	*MAT*α *ric8*Δ::*NAT* recombinant genome	This study
SSK910	Progeny from SSK110 × Bt63	*MAT***a** *ric8*Δ::*NAT* recombinant genome	This study
SSL436	Bt63	*MAT***a** *ric8*Δ::*NEO*	This study
SSL570	SSL436 + SSK110 fusion No.06	*MAT***a**/**a** *NAT NEO*	This study
SSL571	SSL436 + SSK110 fusion No.07	*MAT*α/α *NAT NEO*	This study
SSL576	SSL436 + SSK110 fusion No.12	*MAT*α/α *NAT NEO*	This study
SSL739	SSK910	*MAT***a** *gpa2*Δ::*NEO*_1	This study
SSL740	SSK910	*MAT***a** *gpa2*Δ::*NEO*_2	This study
SSL741	SSK910	*MAT***a** *pka1*Δ::*NEO*_1	This study

Strain Name	Strain background	Genotype	Source
SSL742	SSK910	*MAT***a** *pka1*Δ::*NEO*_2	This study
SSL743	SSK910	*MAT***a** *ste7*Δ::*NEO*_1	This study
SSL744	SSK910	*MAT***a** *ste7*Δ::*NEO*_2	This study
SSL745	SSK910	*MAT***a** *cpk1*Δ::*NEO*_1	This study
SSL746	SSK910	*MAT***a** *cpk1*Δ::*NEO*_2	This study
SSL747	SSK910	*MAT***a** *znf2*Δ::*NEO*_1	This study
SSL748	SSK910	*MAT***a** *znf2*Δ::*NEO*_2	This study
SSL749	SSK910	*MAT***a** *cpa1*Δ::*NEO*_1	This study
SSL750	SSK910	*MAT***a** *cpa1*Δ::*NEO*_2	This study
SSL751	SSK910	*MAT***a** *gpr4*Δ::*NEO*_1	This study
SSL752	SSK910	*MAT***a** *gpr4*Δ::*NEO*_2	This study
SSL753	SSK910	*MAT***a** *mat2*Δ::*NEO*_1	This study
SSL754	SSK910	*MAT***a** *mat2*Δ::*NEO*_2	This study
SSL755	SSK910	*MAT***a** *sxi2*Δ::*NEO*_1	This study
SSL756	SSK910	*MAT***a** *sxi2*Δ::*NEO*_2	This study
SSL757	SSK910	*MAT***a** *spo11*Δ::*NEO*_1	This study
SSL758	SSK910	*MAT***a** *spo11*Δ::*NEO*_2	This study
SSL759	SSK910	*MAT***a** *dmc1*Δ::*NEO*_1	This study
SSL760	SSK910	*MAT***a** *dmc1*Δ::*NEO*_2	This study
YSB25	H99	*MAT*α *gpa2*Δ::*NEO*	(53)

Strain Name	Strain background	Genotype	Source
YSB26	H99	*MAT*α *gpa2*Δ::*NEO*	(53)
YSB85	KN99**a**	*MAT***a** *gpa1*Δ::*NEO*	(71)
YSB86	KN99**a**	*MAT***a** *gpa1*Δ::*NEO*	(71)
YSB137	KN99**a**	*MAT***a** *gpa3*Δ::*NEO*	(53)
YSB138	KN99**a**	*MAT***a** *gpa3*Δ::*NEO*	(53)
SSL808	YSB25 × YSB85	*MAT*α *gpa1*Δ::*NEO*	This study
SSL809	YSB25 × YSB85	*MAT*α *gpa1*Δ::*NEO*	This study
SSL810	YSB25 × YSB85	*MAT***a** *gpa2*Δ::*NEO*	This study
SSL811	YSB25 × YSB85	*MAT***a** *gpa2*Δ::*NEO*	This study
SSL813	YSB25 × YSB85	*MAT*α *gpa1*Δ::*NEO gpa2*Δ::*NEO*	This study
SSL832	SSL813 × YSB137	*MAT*α *gpa3*Δ::*NEO*	This study
SSL833	SSL813 × YSB137	*MAT*α *gpa3*Δ::*NEO*	This study
SSL815	SSL813 × YSB137	*MAT***a** *gpa1*Δ::*NEO gpa2*Δ::*NEO*	This study
SSL816	SSL813 × YSB137	*MAT***a** *gpa1*Δ::*NEO gpa2*Δ::*NEO*	This study
SSL817	SSL813 × YSB137	*MAT*α *gpa1*Δ::*NEO gpa2*Δ::*NEO*	This study
SSL818	SSL813 × YSB137	*MAT*α *gpa1*Δ::*NEO gpa2*Δ::*NEO*	This study
SSL819	SSL813 × YSB137	*MAT***a** *gpa1*Δ::*NEO gpa3*Δ::*NEO*	This study
SSL820	SSL813 × YSB137	*MAT***a** *gpa1*Δ::*NEO gpa3*Δ::*NEO*	This study
SSL821	SSL813 × YSB137	*MAT*α *gpa1*Δ::*NEO gpa3*Δ::*NEO*	This study
SSL822	SSL813 × YSB137	*MAT*α *gpa1*Δ::*NEO gpa3*Δ::*NEO*	This study

Strain Name	Strain background	Genotype	Source
SSL823	SSL813 × YSB137	*MAT***a** *gpa2*Δ::*NEO gpa3*Δ::*NEO*	This study
SSL824	SSL813 × YSB137	*MAT***a** *gpa2*Δ::*NEO gpa3*Δ::*NEO*	This study
SSL825	SSL813 × YSB137	*MAT*α *gpa2*Δ::*NEO gpa3*Δ::*NEO*	This study
SSL831	SSL813 × YSB137	*MAT*α *gpa2*Δ::*NEO gpa3*Δ::*NEO*	This study
SSL827	SSL813 × YSB137	*MAT***a** *gpa1*Δ::*NEO gpa2*Δ::*NEO gpa3*Δ::*NEO*	This study
SSL828	SSL813 × YSB137	*MAT***a** *gpa1*Δ::*NEO gpa2*Δ::*NEO gpa3*Δ::*NEO*	This study
SSL829	SSL813 × YSB137	*MAT*α *gpa1*Δ::*NEO gpa2*Δ::*NEO gpa3*Δ::*NEO*	This study
SSL830	SSL813 × YSB137	*MAT*α *gpa1*Δ::*NEO gpa2*Δ::*NEO gpa3*Δ::*NEO*	This study
YPH106		*MAT***a** *gpa2*Δ::*NEO gpa3*Δ::*NEO*	(53)
YPH118		*MAT***a** *crg1*Δ::*URA5 gpa2*Δ::*NEO gpa3*Δ::*NEO*	(53)
YPH305		*MAT*α *crg2*Δ::*NAT gpa2*Δ::*NEO gpa3*Δ::*NEO*	(53)
YPH308		*MAT*α *gpa2*Δ::*NAT gpa3*Δ::*NEO*	(53)
YPH380		*MAT*α *crg1*Δ::*URA5 gpa2*Δ::*NEO gpa3*Δ::*NEO*	(53)

Supplemental Table S2. Genes deleted in the self-fertile strain.

Gene ID [1]	Gene Name	Encoded protein	Self-fertility	Sporulation
CNAG_04505	*GPA1*	Guanine nucleotide-binding protein subunit α	Slightly delayed	Yes
CNAG_00179	*GPA2*	G protein α subunit	Yes	Yes
CNAG_02090	*GPA3*	G protein α subunit	Yes	Yes
CNAG_06808 [2]	*STE3*	G-protein coupled receptor	Yes	Yes
CNAG_04730	*GPR4*	G-protein coupled receptor	Yes	Yes
n.a. [1]	*SXI2*	Homeodomain (HD) transcription factor	Yes	Yes
CNAG_00396	*PKA1*	Protein kinase A	Slightly delayed	Yes
CNAG_05472	*SPO11*	Meiotic recombination protein	Yes	No
CNAG_07909	*DMC1*	Meiotic recombinase	Yes	No
CNAG_02511	*CPK1*	MAPK	No	n.a.
CNAG_01730	*STE7*	MAPKK	No	n.a.
CNAG_06980 [2]	*STE11*	MAPKKK	No	n.a.
CNAG_06203	*MAT2*	HMG-box transcription factor	No	n.a.
CNAG_03366	*ZNF2*	Master regulator of filamentation, C2H2 type zinc finger	No	n.a.

[1]: The gene IDs are based on the annotation of the *MAT*α H99 genome. *SXI2***a** does not have a gene ID as it is a *MAT***a** specific gene.

[2]: *STE3* and *STE11* are mating-type specific genes, and their *MAT***a** homologs were deleted in the *MAT***a** self-fertile strain SSK910.
