# Supplementary material for: Unisexual reproduction in the global human fungal pathogen Cryptococcus neoformans
Source: bioRxiv. 2025 Sep 2:2025.06.02.657540. Originally published 2025 Jun 3. Preprint. [Version 3] doi: 10.1101/2025.06.02.657540 (PMC12157407; doi:10.1101/2025.06.02.657540)

Supplemental Figure S6

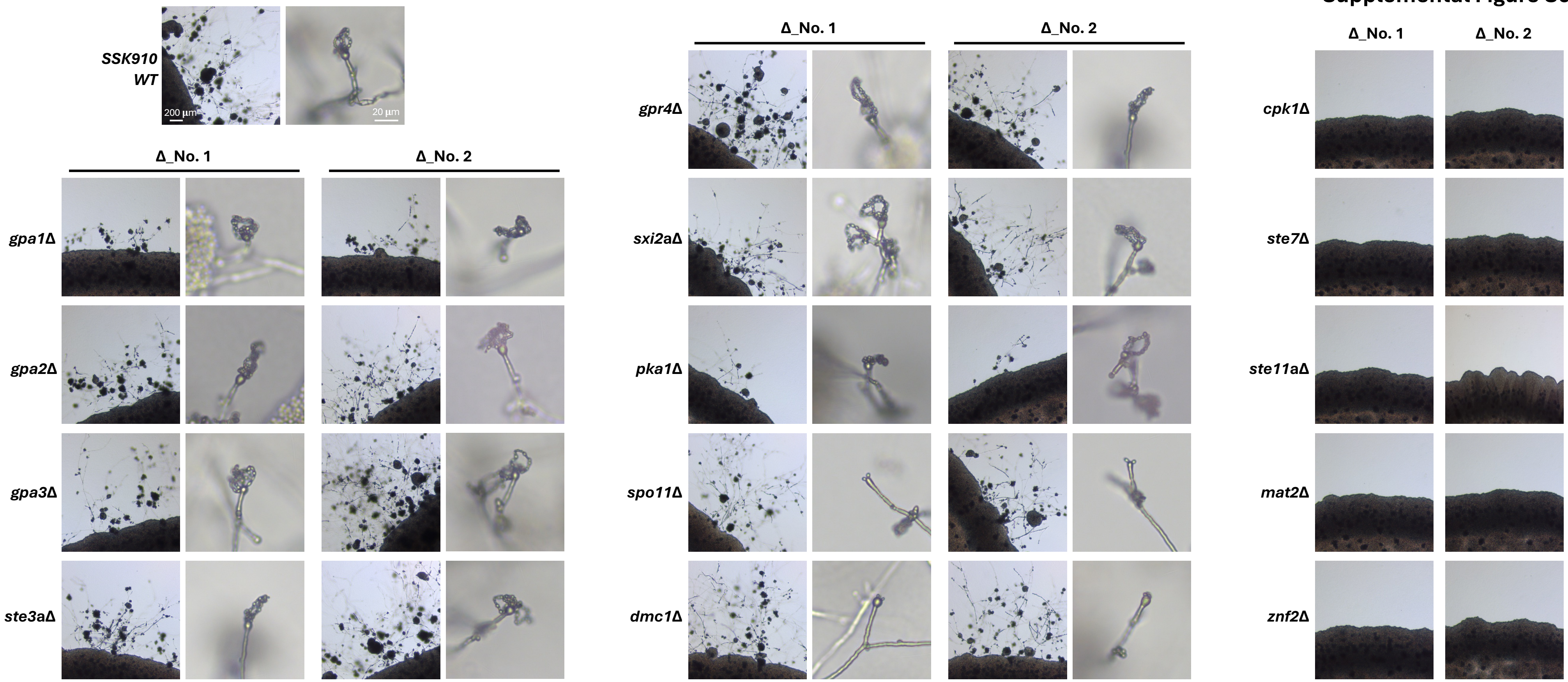

Supplement: Supplement 6 — Supplemental Figure S6. Selfing assay of mutant strains in the self-fertile C. neoformans SSK910 strain background. Light microscopy images of strains with deletions of various genes in the SSK910 background when induced for unisexual reproduction on MS medium. Images shown are from two independent deletion strains of each gene, with images on the right showing the basidia with or without typical basidiospore chains for the deletion strains that were still able to undergo unisexual reproduction. Scales are shown in the images of the SSK910 progenitor strain. [file media-6.pdf]
