# Supplementary material for: Unisexual reproduction in the global human fungal pathogen Cryptococcus neoformans
Source: bioRxiv. 2025 Aug 8:2025.06.02.657540. Originally published 2025 Jun 3. Preprint. [Version 2] doi: 10.1101/2025.06.02.657540 (PMC12157407; doi:10.1101/2025.06.02.657540)
Supplement: Supplementary file 1 [file NIHPP2025.06.02.657540v2-supplement-8.pdf]

bioRxiv preprint doi: https://doi.org/10.1101/2025.06.02.657540; this version posted August 8, 2025. The copyright holder for this preprint (which was not certified by peer review) is the author/funder, who has granted bioRxiv a license to display the preprint in perpetuity. It is made available under aCC-BY 4.0 International license.

Supplemental figure legends

Supplemental Figure S1. Genotypic profiles of the unisexually fertile *C. neoformans* strains. (A) Genotyping of the *MAT* locus for the haploid strains analyzed in this study. (B) Genome analysis of the strains that unisexually reproduce. The blue and red colors indicate SNPs when mapped against the H99 and Bt63 genomes, respectively. For each strain, zoomed-in read coverage maps of chromosome 5 are shown with. the H99 (blue) or Bt63 (red) genome as reference. For strains SSK110 and SSK910, additional zoomed-in maps of SNPs against H99 (blue) or Bt63 (red) along chromosome 5 are included beneath the read-depth plots.

Supplemental Figure S2. Analyses of progeny from diploid *MAT*a/a *C. neoformans* strain SSL570.

Variant mapping of the parental strain SSL570 and its dissected basidiospore chains. For the parental strain SSL570, the variants against H99 (blue) and Bt63 (red) were mapped against the H99 and Bt63 genomes, respectively. For the progeny, the variants against H99 and Bt63 were all mapped with the H99 genome as reference. The purple column indicates the location of the *MAT* locus.

Supplemental Figure S3. Analyses of progeny from diploid *MAT*α/α *C. neoformans* strain SSL571.

Variant mapping of the parental strain SSL571 and its dissected basidiospore chains. For the parental strain SSL571, the variants against H99 (blue) and Bt63 (red) were mapped against the H99 and Bt63 genomes, respectively. For the progeny, the variants against H99 and Bt63 were all mapped with the H99 genome as reference. The purple column indicates the location of the *MAT* locus.

Supplemental Figure S4. Analyses of progeny from diploid *MAT*α/α *C. neoformans* strain SSL576.

Variant mapping of the parental strain SSL576 and its dissected basidiospore chains. For the parental strain SSL576, the variants against H99 (blue) and Bt63 (red) were mapped against the H99 and Bt63 genomes, respectively. For the progeny, the variants against H99 and Bt63 were

bioRxiv preprint doi: https://doi.org/10.1101/2025.06.02.657540; this version posted August 8, 2025. The copyright holder for this preprint (which was not certified by peer review) is the author/funder, who has granted bioRxiv a license to display the preprint in perpetuity. It is made available under aCC-BY 4.0 International license.

all mapped with the H99 genome as reference. The purple column indicates the location of the *MAT* locus.

Supplemental Figure S5. PCR validation of the deletion strains in the self-fertile SSK910 strain background.

Each image demonstrates PCR validation of two independent deletion strains of one gene (labeled at the right bottom corner of the image), including 5'- and 3'- junction PCRs (left and right panels, respectively, amplifying only from deletion strains) and internal PCR (middle panel, amplifying only from wild-type controls). For each PCR reaction, samples from left to right are: deletion strain No.1, deletion strain No.2, KN99a wild-type control, KN99α wild-type control, and ddH_2O negative PCR control.

Supplemental Figure S6. Selfing assay of mutant strains in the self-fertile *C. neoformans* SSK910 strain background.

Light microscopy images of strains with deletions of various genes in the SSK910 background when induced for unisexual reproduction on MS medium. Images shown are from two independent deletion strains of each gene, with images on the right showing the basidia with or without typical basidiospore chains for the deletion strains that were still able to undergo unisexual reproduction. Scales are shown in the images of the SSK910 progenitor strain.

Supplemental Table S1. Strains analyzed in this study.

Strain Name	Strain background	Genotype	Source
KN99α	n.a.	*MAT*α wildtype	(70)
KN99**a**	n.a.	*MAT***a** wildtype	(70)
AD1-7a	n.a.	*MAT*α wildtype	(19)
T4	n.a.	*MAT*α wildtype	(19)
Bt63	n.a.	*MAT***a** wildtype	(19)
Bt65	n.a.	*MAT***a** wildtype	(19)
SSI867	KN99α	*MAT*α *ric8*Δ::*NAT*	(27)
SSI868	KN99α	*MAT*α *ric8*Δ::*NAT*	(27)
SSI869	Progeny from SSI867 × KN99**a**	*MAT***a** *ric8*Δ::*NAT*	(27)
SSI870	Progeny from SSI868 × KN99**a**	*MAT***a** *ric8*Δ::*NAT*	(27)
SSK110	Progeny from SSI869 × AD1-7a	*MAT*α *ric8*Δ::*NAT* recombinant genome	This study
SSK910	Progeny from SSK110 × Bt63	*MAT***a** *ric8*Δ::*NAT* recombinant genome	This study
SSL436	Bt63	*MAT***a** *ric8*Δ::*NEO*	This study
SSL570	SSL436 + SSK110 fusion No.06	*MAT***a**/**a** *NAT NEO*	This study
SSL571	SSL436 + SSK110 fusion No.07	*MAT*α/α *NAT NEO*	This study
SSL576	SSL436 + SSK110 fusion No.12	*MAT*α/α *NAT NEO*	This study
SSL739	SSK910	*MAT***a** *gpa2*Δ::*NEO*_1	This study
SSL740	SSK910	*MAT***a** *gpa2*Δ::*NEO*_2	This study
SSL741	SSK910	*MAT***a** *pka1*Δ::*NEO*_1	This study

Strain Name	Strain background	Genotype	Source
SSL742	SSK910	*MAT***a** *pka1*Δ::*NEO*_2	This study
SSL743	SSK910	*MAT***a** *ste7*Δ::*NEO*_1	This study
SSL744	SSK910	*MAT***a** *ste7*Δ::*NEO*_2	This study
SSL745	SSK910	*MAT***a** *cpk1*Δ::*NEO*_1	This study
SSL746	SSK910	*MAT***a** *cpk1*Δ::*NEO*_2	This study
SSL747	SSK910	*MAT***a** *znf2*Δ::*NEO*_1	This study
SSL748	SSK910	*MAT***a** *znf2*Δ::*NEO*_2	This study
SSL749	SSK910	*MAT***a** *cpa1*Δ::*NEO*_1	This study
SSL750	SSK910	*MAT***a** *cpa1*Δ::*NEO*_2	This study
SSL751	SSK910	*MAT***a** *gpr4*Δ::*NEO*_1	This study
SSL752	SSK910	*MAT***a** *gpr4*Δ::*NEO*_2	This study
SSL753	SSK910	*MAT***a** *mat2*Δ::*NEO*_1	This study
SSL754	SSK910	*MAT***a** *mat2*Δ::*NEO*_2	This study
SSL755	SSK910	*MAT***a** *sxi2*Δ::*NEO*_1	This study
SSL756	SSK910	*MAT***a** *sxi2*Δ::*NEO*_2	This study
SSL757	SSK910	*MAT***a** *spo11*Δ::*NEO*_1	This study
SSL758	SSK910	*MAT***a** *spo11*Δ::*NEO*_2	This study
SSL759	SSK910	*MAT***a** *dmc1*Δ::*NEO*_1	This study
SSL760	SSK910	*MAT***a** *dmc1*Δ::*NEO*_2	This study
YSB25	H99	*MAT*α *gpa2*Δ::*NEO*	(61)

Strain Name	Strain background	Genotype	Source
YSB26	H99	*MAT*α *gpa2*Δ::*NEO*	(61)
YSB85	KN99**a**	*MAT***a** *gpa1*Δ::*NEO*	(71)
YSB86	KN99**a**	*MAT***a** *gpa1*Δ::*NEO*	(71)
YSB137	KN99**a**	*MAT***a** *gpa3*Δ::*NEO*	(61)
YSB138	KN99**a**	*MAT***a** *gpa3*Δ::*NEO*	(61)
SSL808	YSB25 × YSB85	*MAT*α *gpa1*Δ::*NEO*	This study
SSL809	YSB25 × YSB85	*MAT*α *gpa1*Δ::*NEO*	This study
SSL810	YSB25 × YSB85	*MAT***a** *gpa2*Δ::*NEO*	This study
SSL811	YSB25 × YSB85	*MAT***a** *gpa2*Δ::*NEO*	This study
SSL813	YSB25 × YSB85	*MAT*α *gpa1*Δ::*NEO gpa2*Δ::*NEO*	This study
SSL832	SSL813 × YSB137	*MAT*α *gpa3*Δ::*NEO*	This study
SSL833	SSL813 × YSB137	*MAT*α *gpa3*Δ::*NEO*	This study
SSL815	SSL813 × YSB137	*MAT***a** *gpa1*Δ::*NEO gpa2*Δ::*NEO*	This study
SSL816	SSL813 × YSB137	*MAT***a** *gpa1*Δ::*NEO gpa2*Δ::*NEO*	This study
SSL817	SSL813 × YSB137	*MAT*α *gpa1*Δ::*NEO gpa2*Δ::*NEO*	This study
SSL818	SSL813 × YSB137	*MAT*α *gpa1*Δ::*NEO gpa2*Δ::*NEO*	This study
SSL819	SSL813 × YSB137	*MAT***a** *gpa1*Δ::*NEO gpa3*Δ::*NEO*	This study
SSL820	SSL813 × YSB137	*MAT***a** *gpa1*Δ::*NEO gpa3*Δ::*NEO*	This study
SSL821	SSL813 × YSB137	*MAT*α *gpa1*Δ::*NEO gpa3*Δ::*NEO*	This study
SSL822	SSL813 × YSB137	*MAT*α *gpa1*Δ::*NEO gpa3*Δ::*NEO*	This study

Strain Name	Strain background	Genotype	Source
SSL823	SSL813 × YSB137	*MAT***a** *gpa2*Δ::*NEO gpa3*Δ::*NEO*	This study
SSL824	SSL813 × YSB137	*MAT***a** *gpa2*Δ::*NEO gpa3*Δ::*NEO*	This study
SSL825	SSL813 × YSB137	*MAT*α *gpa2*Δ::*NEO gpa3*Δ::*NEO*	This study
SSL831	SSL813 × YSB137	*MAT*α *gpa2*Δ::*NEO gpa3*Δ::*NEO*	This study
SSL827	SSL813 × YSB137	*MAT***a** *gpa1*Δ::*NEO gpa2*Δ::*NEO gpa3*Δ::*NEO*	This study
SSL828	SSL813 × YSB137	*MAT***a** *gpa1*Δ::*NEO gpa2*Δ::*NEO gpa3*Δ::*NEO*	This study
SSL829	SSL813 × YSB137	*MAT*α *gpa1*Δ::*NEO gpa2*Δ::*NEO gpa3*Δ::*NEO*	This study
SSL830	SSL813 × YSB137	*MAT*α *gpa1*Δ::*NEO gpa2*Δ::*NEO gpa3*Δ::*NEO*	This study

Supplemental Table S2. Primers used in this study.

(Please see the attached Excel file)
