# Supplementary material for: Unisexual reproduction in the global human fungal pathogen Cryptococcus neoformans
Source: bioRxiv. 2025 Jun 3:2025.06.02.657540. Preprint. [Version 1] doi: 10.1101/2025.06.02.657540 (PMC12157407; doi:10.1101/2025.06.02.657540)
Supplement: Supplementary file 1 [file NIHPP2025.06.02.657540v1-supplement-6.pdf]

Supplemental Table S1. Strains analyzed in this study.

Strain Name	Strain background	Genotype	Source
KN99α	n.a.	*MAT*α wildtype	
KN99**a**	n.a.	*MAT***a** wildtype	
AD1-7a	n.a.	*MAT*α wildtype	
T4	n.a.	*MAT*α wildtype	
Bt63	n.a.	*MAT***a** wildtype	
Bt65	n.a.	*MAT***a** wildtype	
SSI867	KN99α	*MAT*α *ric8*Δ::*NAT*	This study
SSI868	KN99α	*MAT*α *ric8*Δ::*NAT*	This study
SSI869	Progeny from SSI867 × KN99**a**	*MAT***a** *ric8*Δ::*NAT*	This study
SSI870	Progeny from SSI868 × KN99**a**	*MAT***a** *ric8*Δ::*NAT*	This study
SSK110	Progeny from SSI869 × AD1-7a	*MAT*α *ric8*Δ::*NAT* recombinant genome	This study
SSK910	Progeny from SSK110 × Bt63	*MAT***a** *ric8*Δ::*NAT* recombinant genome	This study
SSL436	Bt63	*MAT***a** *ric8*Δ::*NEO*	This study
SSL570	SSL436 + SSK110 fusion No.06	*MAT***a**/**a** *NAT NEO*	This study
SSL571	SSL436 + SSK110 fusion No.07	*MAT*α/α *NAT NEO*	This study
SSL576	SSL436 + SSK110 fusion No.12	*MAT*α/α *NAT NEO*	This study
