# Supplementary material for: Unisexual reproduction in the global human fungal pathogen Cryptococcus neoformans
Source: bioRxiv. 2025 Jun 3:2025.06.02.657540. Preprint. [Version 1] doi: 10.1101/2025.06.02.657540 (PMC12157407; doi:10.1101/2025.06.02.657540)

Supplemental Figure S5

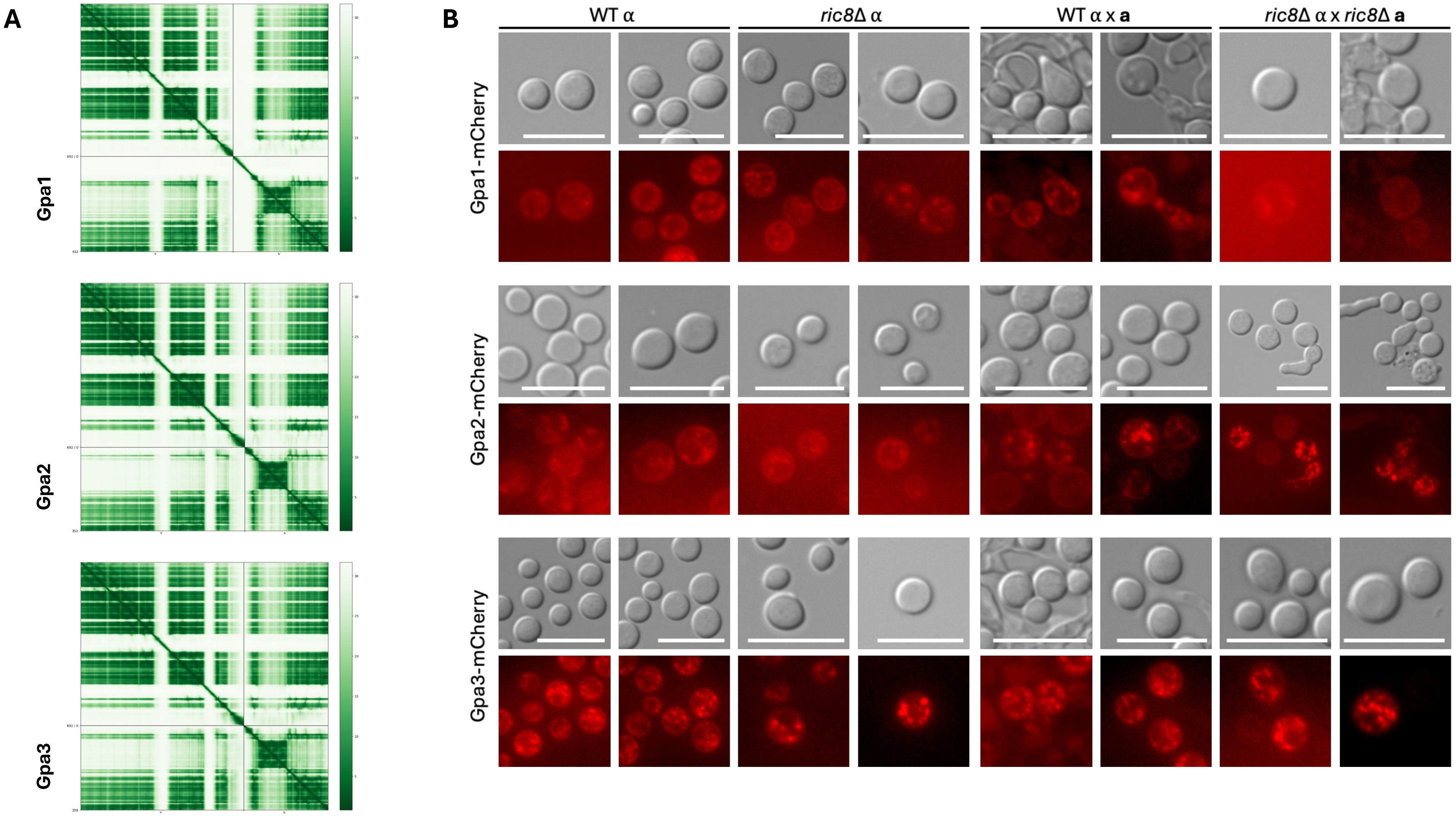

Supplement: Supplementary file 6 [file media-5.pdf]
